# Antiarrhythmic drugs in out-of-hospital cardiac arrest: is there a place for potassium chloride?

**DOI:** 10.1186/s13054-017-1732-z

**Published:** 2017-06-17

**Authors:** Romain Jouffroy, Benoît Vivien

**Affiliations:** SAMU de Paris, Service d’Anesthésie Réanimation, Hôpital Necker—Enfants Malades, Assistance Publique—Hôpitaux de Paris, and Université Paris Descartes—Paris 5, Paris, France

In the March 2017 issue of *Critical Care*, Tagami et al. [1] emphasized that amiodarone, lidocaine, and nifekalant similarly improve survival at hospital admission for out-of-hospital cardiac arrest (OHCA) patients due to refractory ventricular fibrillation (VF). However, neither amiodarone nor lidocaine has been shown to improve long-term survival or neurologic recovery [2].

During resuscitation, the main objective of cardiopulmonary resuscitation is to maintain blood flow pending etiological treatment, and to try and restore sinus rhythm [3]. Antiarrhythmic drugs work on sodium, calcium, and/or potassium channels, and therefore modify the defibrillation threshold [1]. Amiodarone induces a prolonged hypotension, this effect being mainly linked to its solvent, whereas lidocaine causes a concentration-dependent increase in defibrillation energy requirements and a negative inotropic effect. However, according to current guidelines, antiarrhythmic drugs should be administered during the metabolic phase of cardiac arrest, which is paradoxically the most unfavorable phase for their efficiency [3].

Potassium chloride (KCl) is the major component of cardioplegia solutions used to arrest the heart during cardiac surgery under extracorporeal life support (ECLS). This effect is fully reversible, enabling return of spontaneous circulation after its administration. Twenty-five years ago, Beyersdorf et al. [4] observed that administration of a high-potassium solution could salvage cardiac arrest patients due to an irreversible VF. This report suggested that KCl injection could be an alternative to conventional antiarrhythmic drugs during the metabolic phase of cardiac arrest due to irreversible VF. As evidence, direct intravenous KCl injection was recently shown efficient to stop refractory VF in a patient under continuous ECLS: a few minutes after KCl administration, the heart recovered a hemodynamically efficient normal sinus rhythm [5].

In cardiac arrest due to refractory VF, the proposition of direct intravenous KCl injection can primarily be surprising, because there is a suspected ischemia–reperfusion syndrome with metabolic acidosis and thus hyperkalemia, underlying the use of the pure potassium blocker nifekalant [1]. Conversely, we must keep in mind that medical resuscitation using a beta-agonist drug (epinephrine) and sodium bicarbonate can induce transfer hypokalemia. Moreover, the latter can be deleterious on membrane stabilization, and can consecutively sustain ventricular dysrhythmia. Elsewhere, unlike amiodarone or lidocaine, KCl administration is not responsible for a persistent negative inotropic effect.

Investigating the efficacy of direct intravenous KCl injection for OHCA due to irreversible VF seems therefore to be a relevant research pathway, to define the potential benefit of this treatment in the therapeutic strategy as compared with conventional antiarrhythmic drugs.

## Abbreviations

ECLS: Extracorporeal life support
KCl: Potassium chloride
OHCA: Out-of-hospital cardiac arrest
VF: Ventricular fibrillation
